# The impact of COVID-19 on healthcare workers: risk factors, sources of infection, and sickness absenteeism

**DOI:** 10.55730/1300-0144.6159

**Published:** 2025-08-19

**Authors:** Ezgi GÜLTEN, Güle ÇINAR, İrem AKDEMİR, Elif Mukime SARICAOĞLU, İlkay TAŞDAN, Emine ATMACA, Sibel KAYMAKÇI, Fatma ŞANAL, Dilek Sevim AKDAŞ, Arzu BÜYÜM, Gülşah KOCAALİ, Safiye TARAKÇI, Atilla Halil ELHAN, Mehmet Serhat BİRENGEL, Kemal Osman MEMİKOĞLU, Alpay AZAP

**Affiliations:** 1 Department of Infectious Diseases and Clinical Microbiology, Faculty of Medicine, Ankara University, Ankara, Turkiye; 2 Infection Control Committee, Ankara University Hospitals, Ankara, Turkiye; 3 Department of Biostatistics, Faculty of Medicine, Ankara University, Ankara, Turkiye

**Keywords:** COVID-19, healthcare workers, pandemic preparedness, sickness absenteeism, vaccination coverage

## Abstract

**Background/aim:**

Healthcare workers (HCWs) face increased risks of COVID-19 infection due to occupational exposure. Understanding infection sources, risk factors, and workforce losses is crucial for mitigating these impacts in future pandemics. This study aimed to evaluate the risk factors, infection sources, and sickness absenteeism among HCWs diagnosed with SARS-CoV-2 infection, providing insights to enhance infection control strategies.

**Materials and methods:**

The study included 2153 HCWs diagnosed with SARS-CoV-2 between March 2020 and February 2023 at a tertiary care hospital in Türkiye. Demographic, clinical, and professional characteristics, personal protective equipment (PPE) usage, infection sources, and vaccination data were analyzed. Statistical analyses were conducted using the R programming language.

**Results:**

Among infected HCWs, 68.5% were female, and nurses accounted for 39.4% of cases. PPE compliance was significantly higher in COVID-19-specific units compared to non-COVID-19 units (p < 0.001), yet inappropriate PPE use was observed in 10.5% of exposures. Infections were community-acquired in 35.4%, hospital-acquired from colleagues in 24.9%, and from patients in 7.2% of cases, while 32.5% of infections had unidentified sources. Vaccination data revealed that 73.7% of HCWs missed opportunities for timely vaccination, and nearly half of the vaccinated HCWs were not within the protective window at the time of infection. Sickness absenteeism totaled 23,454 days, exceeding expected workforce loss by 2891 days. Hospitalization occurred in 4.1% of cases, with one fatality.

**Conclusion:**

The findings highlight the critical importance of comprehensive infection control measures, improved vaccination uptake, and robust workforce management to protect HCWs and sustain healthcare systems during pandemics.

## 1. Introduction

Healthcare workers (HCWs) are at increased risk of contracting infections such as coronavirus disease 2019 (COVID-19), influenza, and tuberculosis due to their occupational environments. The elevated risk of transmission during aerosol-generating procedures, combined with the ability of these infections to spread via both droplets and aerosols, highlights the critical importance of proper personal protective equipment (PPE) usage [1–3]. Inadequate implementation of infection control measures poses serious threats to HCWs’ health and contributes to the increased spread of nosocomial infections [4].

The severe acute respiratory syndrome coronavirus 2 (SARS-CoV-2) pandemic has profoundly altered life, work, and communication while causing substantial disruption to healthcare systems worldwide [5]. According to data from the World Health Organization (WHO), as of January 12, 2025, 777,315,739 individuals had been infected with SARS-CoV-2, resulting in 7,083,869 deaths^1^. Healthcare workers have borne the dual burden of the pandemic, simultaneously navigating the social and economic challenges the broader community faces and the profound difficulties of saving lives in high-risk healthcare settings [5]. Furthermore, the increased risk of COVID-19 infection among HCWs, stemming from occupational exposure and indirectly affecting their close contacts, has been recognized as a significant factor contributing to the deterioration of their mental health [6,7].

The notion that the 21st century could be the “pandemic century” is increasingly accepted by scientists and public health experts. Factors such as globalization, rapid urbanization, increased travel frequency, population density, and climate change are anticipated to increase the prevalence and spread of infectious diseases [8,9]. This situation highlights the critical need for comprehensive pandemic preparedness strategies. Therefore, sharing the lessons learned from the COVID-19 pandemic is pivotal in enhancing preparedness for future pandemics.

This study aims to contribute to the prevention of nosocomial transmission and workforce loss among frontline HCWs during future pandemics by examining strategies for protecting HCWs from SARS-CoV-2 infection, ensuring timely diagnosis, identifying hospital contacts of infected HCWs, tracing infection sources, assessing HCWs’ attitudes towards preventive measures, and evaluating work absence due to COVID-19.

## 2. Materials and methods

### 2.1. Study design and setting

The hospitals of xxxx provide tertiary healthcare services with a capacity of 1528 inpatient beds and 192 intensive care unit (ICU) beds across two campuses, with 7000 HCWs.

At our hospitals, the first patient confirmed via real-time polymerase chain reaction (RT-PCR) testing was detected on March 17, 2020. In our institution, a “COVID-19 Commission” was established in February 2020 under the leadership and initiative of the infection control team, which comprises infectious diseases and clinical microbiology specialists and infection control nurses in collaboration with hospital administrators, microbiology specialists, pulmonologists, and intensive care specialists. In accordance with the commission’s decision, all relevant healthcare personnel were included in the workforce pool, to be mobilized for COVID-19 wards and intensive care units. Resident physicians, nurses, patient care assistants, and cleaning staff worked in rotating eight-hour shifts for three-week periods. Infectious diseases and clinical microbiology specialists, pulmonologists, pediatricians, and intensive care unit specialists adhered to fixed eight-hour shifts on weekdays, while providing rotational coverage during weekends and after-hours for both inpatient wards and outpatient clinics. A similar working pattern was implemented in the COVID-19 outpatient clinics.

Prior to the initiation of COVID-19 case management in our institution, all HCWs were provided with training on COVID-19 transmission and prevention methods. For those assigned to COVID-19 units, the training was repeated before commencing their duties. The training content was reviewed and updated each time based on the latest COVID-19-related information. Visual reminders promoting the use of PPEs were placed throughout all inpatient wards, outpatient clinics, and administrative units. Accordingly, the use of surgical masks was recommended and encouraged in all outpatient clinics, inpatient wards, and administrative units, while N95/FFP2 masks were advised for all intensive care units and operating rooms. Additionally, the specific PPE required for various interventions and procedures was determined based on WHO recommendations^2^.

#### 2.1.1. Measures undertaken to ensure the early detection of SARS-CoV-2 infection in HCWs and to prevent in-hospital transmission

##### 2.1.1.1. PPE compliance monitoring

**Figure 1:**
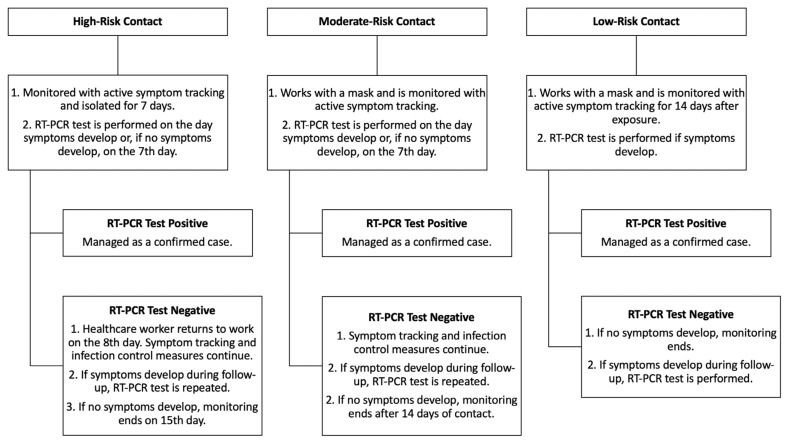
Follow-up measures for HCWs in case of inadequate use of personal protective equipments.

Compliance with PPE usage was audited through intensified field inspections by the infection control team and department supervisors during the pandemic. In case of inadequate use of PPE was identified during patient care, treatment, or procedures involving confirmed or suspected COVID-19 cases, follow-up measures for HCWs were implemented (Figure 1).

##### 2.1.1.2. Daily symptom screening

Supervisors in all departments were tasked with conducting daily symptom assessments of HCWs and maintaining records. HCWs presenting with COVID-19-related symptoms were referred to the COVID-19 outpatient clinic for further evaluation.

##### 2.1.1.3. Investigation of the source of infection and identification of in-hospital contacts

The contact persons to be informed in case of SARS-CoV-2 infection among HCWs were formally announced to all units. To prevent in-hospital transmission of COVID-19, active contact tracing was conducted by the infection control team for HCWs with confirmed SARS-CoV-2 infections (via oronasopharyngeal RT-PCR testing) between March 17, 2020, and February 1, 2023. Following the immediate isolation of infected HCWs, a series of telephone interviews was conducted to investigate the source of infection and identify in-hospital contacts. These findings were recorded in the case documentation form (Supplementary material 1). Since the first infected HCW was reported to the infection control team on April 7, 2020, flowcharts detailing risk categories, and infection source determination have been provided in Table 1, and Table 2, respectively.

### 2.2. Recorded outcomes

Data collected during interviews with SARS-CoV-2-infected HCWs encompassed demographic, clinical, and professional characteristics, including age, gender, RT-PCR test dates, symptom duration, workplace details, PPE use, contact history, vaccination status, hospitalization needs, and sickness absenteeism. In Türkiye, routine analyses of SARS-CoV-2 variants were not performed. Variant identification was based on data from GISAID research during respective periods^3^. HCWs in Türkiye were prioritized for COVID-19 vaccination. In January 2021, the CoronaVac vaccine, and in June 2021, the Pfizer-BNT162b2 mRNA-based vaccine were administered to HCWs in two doses, with a four-week interval. In January and July 2022, and January 2023, HCWs were advised to receive booster doses of either CoronaVac or Pfizer-BNT162b2 mRNA-based vaccines by national health authorities^4^. However, any form of the updated mRNA vaccines, which protect against the Omicron variants, have never been available. Vaccination data of HCWs were classified as follows:

#### 2.2.1. Primary vaccination scheme

Completion of two doses of either CoronaVac or Pfizer-BNT162b2 mRNA-based vaccine at four-week intervals.

#### 2.2.2. Booster dose

Administration of an additional dose following the primary scheme.

#### 2.2.3. Protective window

Protective window refers to “a minimum of 14 days and a maximum of 180 days after the primary vaccination scheme” and/or “a minimum of 14 days and a maximum of 180 days after completing a second primary scheme with a different vaccine type”.

#### 2.2.4. Vaccination opportunity

This refers to HCWs receiving the available vaccine option in the country during the specified period. It is expected that HCWs were vaccinated within the first month following the month the vaccine became available.

#### 2.2.5. Duration of isolation

Isolation durations for COVID-19 cases, as determined by the Turkish Ministry of Health, were implemented for HCWs in our hospitals. These durations were set at 14 days from the start of the pandemic until October 2021, 10 days between October 2021 and January 2022, and seven days from January 2022 onward^4^. In this study, the number of days of sickness absenteeism and related evaluations were made based on these durations.

### 2.3. Statistical analysis

For statistical analysis, the R programming language (version 4.2.3) was used. Categorical variables were expressed as numbers and percentages, while continuous variables were summarized as means (±standard deviation) or medians (minimum–maximum). Comparisons of categorical variables between groups were conducted using the Chi-square test, and differences in nonnormally distributed continuous variables between two or more groups were evaluated using the Mann-Whitney U test and Kruskal-Wallis variance analysis, respectively. A p-value of < 0.05 was considered statistically significant.

### 2.4. Ethical approval

This study was conducted in accordance with the principles of the Declaration of Helsinki and was approved by the Human Research Ethics Committee of the …. on October 17, 2024 with decision number 2024/631.

## 3. Results

**Figure 2:**
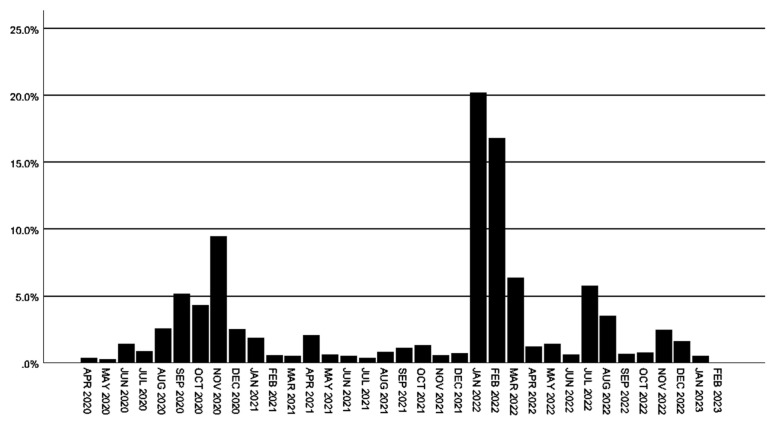
Monthly distribution of the number of SARS-CoV-2-infected HCWs.

In our study, 2153 HCWs diagnosed with SARS-CoV-2 infection between March 17, 2020, and February 1, 2023, were evaluated. According to the data, 408 (19%) and 797 (37%) HCWs were infected during the periods of September–October–November 2020 and January–February 2022, respectively. Monthly distribution of the number of SARS-CoV-2 infected HCWs was provided in Figure 2 and Supplementary Material 2.

Among the SARS-CoV-2-infected HCWs, 1474 (68.5%) were female, with a mean age of 36 years (± 9.6) and an average professional experience of 11.7 years (±9.6). Nurses accounted for 849 (39.4%) of all infected HCWs. It was determined that 285 (13.2%) of the SARS-CoV-2-infected HCWs were employed in COVID-19-specific units (e.g., COVID-19 outpatient clinics, emergency services, COVID-19 inpatient wards, COVID-19 ICUs) at the time of infection. The demographic and occupational characteristics of HCWs infected with SARS-CoV-2 are summarized in Table 3.

Healthcare workers identified by the infection control team and unit supervisors as having inappropriate PPE use during contact with COVID-19 patients were followed up according to the protocol outlined in Figure 1. Of the 121 HCWs identified, 76 (62.8%), 34 (28.1%), and 11 (9.1%) were categorized as low, medium, and high risk, respectively. Among those in the low-risk category, no COVID-19-related symptoms developed during the follow-up period. In contrast, five (14.7%) of the 34 workers in the medium-risk category and three (27.3%) of the 11 workers in the high-risk category tested positive for SARS-CoV-2 via oronasopharyngeal RT-PCR after the related exposure.

Among HCWs diagnosed with SARS-CoV-2 infection while working in COVID-19-specific units, 18 (6.3%) were found to have inappropriate use of PPE. Of the 1868 HCWs detected to be infected while working in non-COVID-19 units, 468 were in units with hospitalized patients who were later identified as SARS-CoV-2-positive. Among these, 61 (13%) were determined to have used PPE inappropriately. Overall, among 753 HCWs exposed to confirmed or suspected COVID-19 cases, 79 (10.5%) failed to use PPEs appropriately. Consequently, the rate of appropriate PPE use was significantly higher in COVID-19-specific units compared to non-COVID-19 units (p < 0.001).

The sources of SARS-CoV-2 infection were evaluated as summarized in Table 1. The source of infection could not be identified for 699 (32.5%) of the infected HCWs. It was determined that 762 (35.4%) and 692 (32.1%) acquired SARS-CoV-2 infection from community (non-hospital) and hospital settings, respectively. Among the cases attributed to hospital-acquired infection, 536 (24.9%) were linked to workplace exposure from colleagues, and 156 (7.2%) were associated with exposure to patients in the unit where they worked. The infection source for HCWs with identified sources was further evaluated based on their unit of work (Table 4). HCWs in non-COVID-19 units were more likely to acquire infection from community settings and workplace exposure to colleagues compared to those in COVID-19-specific units (p < 0.001).

The contact between HCWs infected with SARS-CoV-2 and other hospital staff, as well as the risk category of the contact, was evaluated according to Table 1 and Figure 1. It was determined that 1566 (72.7%) HCWs had no risky contact with other hospital staff. However, 587 HCWs were found to have had contact with 1685 other HCWs, categorized as low-risk (257 contacts), medium-risk (591 contacts), and high-risk (837 contacts). Among HCWs with low-risk contact, no COVID-19-related symptoms developed during the follow-up period. In contrast, 77 (13%) of 591 workers with medium-risk contact and 203 (24.3%) of 837 workers with high-risk contact tested positive for SARS-CoV-2 via oronasopharyngeal RT-PCR during follow-up.

The findings of this study revealed that 621 (28.8%) HCWs were infected with SARS-CoV-2 during the period when COVID-19 vaccines were not yet available in Türkiye. Of the remaining 1532 (71.2%) HCWs, 122 (7.9%) missed the opportunity to complete the primary vaccination schedule. Among the 1410 HCWs who completed the primary vaccination schedule, 700 (49.6%) were not within the protective window at the time of infection. Furthermore, among the 1532 HCWs infected during the vaccine-available period, 1129 (73.7%) missed the vaccination opportunity available for that period.

A total of 89 (4.1%) HCWs required hospitalization due to SARS-CoV-2 infection, with an average hospital stay of 8.1 days (±4.9). Of these, two HCWs (0.1%) required admission to the intensive care unit (ICU), and one HCW (0.05%) lost his life. In total, 2152 SARS-CoV-2-infected HCWs were absent from work for a total of 23,454 days. Duration of sickness absenteeism was categorized according to the Turkish Ministry of Health’s isolation recommendations (7, 10, and 14 days) during different phases of the pandemic, and the expected, actual, and unforeseen workforce loss was evaluated as summarized in Table 5.

## 4. Discussion

Healthcare workers worldwide have been among the groups most vulnerable to acquiring SARS-CoV-2 infections throughout the COVID-19 pandemic [10]. Understanding COVID-19 risk factors among HCWs is critical for formulating effective preventive strategies and policies to protect this essential workforce from the devastating health impacts of the pandemic. Accordingly, this study presents the results of initiatives implemented in our institution to protect HCWs from SARS-CoV-2 infection, ensure timely diagnosis, identify hospital contacts of infected HCWs, trace sources of infection, assess HCWs’ approaches to protective measures, and evaluate sickness absenteeism due to COVID-19.

In our study, the two prominent peak periods of SARS-CoV-2 infection among HCWs can be attributed to distinct factors. The first peak, observed between September and November 2020, occurred prior to the identification of the Alpha variant (B.1.1.7) and was likely driven by the relaxation of social isolation measures in the country^5^. The second peak, seen between January and February 2022, coincided with the circulation of the Omicron variant (B.1.1.529), as reported by GISAID studies, and the implementation of shorter isolation durations by national and international authorities^4^,^6^. These shortened isolation periods, coupled with the prolonged viability of the virus, as noted in the literature, may have facilitated increased transmission within hospital settings. Supporting this, Keske et al. [11] reported that viral cultures remained positive on day seven in 52% of nonsevere COVID-19 cases infected with the Omicron variant, highlighting the potential role of these factors in the latter peak.

Numerous studies have highlighted that most HCWs infected with SARS-CoV-2 are female and nurses [12,13]. Our findings align with these data. The predominance of women among infected HCWs is primarily due to the higher representation of women in the healthcare settings. According to WHO reports, 67% of HCWs worldwide are women, with many serving as nurses or caregivers^7^. Nurses, who have direct, prolonged, and close contact with patients, are particularly vulnerable to infection due to their intense occupational exposure [13].

Identifying the sources of infection among HCWs during the pandemic is crucial for understanding transmission pathways and developing preventive measures. In our study, the source of infection could not be identified in 32.5% of cases. Shaw et al. [14] reported this rate as 58%. This inability to identify the infection source reflects the complexity of nosocomial and community transmission and highlights the risks HCWs face both at work and in their private lives. Research conducted since the onset of the COVID-19 pandemic has demonstrated that SARS-CoV-2 infection among HCWs can originate from both hospital and community settings [14–16]. In the studies mentioned, these rates have varied widely, ranging from 15.1% to 83% and 17% to 42.7%, respectively, depending on the social isolation measures in place during the periods when the studies were conducted [14–16]. In our study, 32.1% and 35.4% of HCWs acquired SARS-CoV-2 infection from hospital and community sources, respectively. These findings emphasize the need for strict implementation of infection control measures both within hospitals and in the community.

In our study, 7.2% of HCWs were infected due to occupational exposure. In a study evaluating only SARS-CoV-2 infected HCWs with identifiable infection source, this rate was reported as 52%. However, in studies evaluating all cases, the rates have been reported to range from 3.55% to 53.6% [14–17]. Consistent with the literature, our findings show that hospital-acquired infections among HCWs most commonly occurred through contact with colleagues [14–17]. Additionally, HCWs in non-COVID-19 units had lower compliance with PPE usage and were more likely to acquire SARS-CoV-2 infection from community settings and colleagues. This is likely related to the professional and social precautions taken by HCWs in COVID-19 units to protect themselves and their families. The lower compliance with PPE use among HCWs in non-COVID-19 units is thought to increase the risk of infection from community. These findings underscore the necessity of promoting infection control culture across all hospital units. It is noteworthy that 14.7% and 13% of HCWs categorized as medium risk for occupational and non-occupational in-hospital exposures, respectively, were found to have contracted COVID-19. To better prepare for future pandemics, we propose the implementation of additional measures aimed at reducing hospital transmission. These include: conducting more frequent training on infection control practices; enhancing early detection of infection; reevaluating exposure risk categories; optimizing staff flow to minimize crowding in shared areas; adopting digital platforms for meetings and collaboration; redesigning shared spaces such as rest and dining areas; installing impermeable partitions in common areas; improving indoor ventilation systems; increasing the use of contactless technologies; and restricting entry and exit points.

The low vaccination rates among HCWs during the COVID-19 pandemic are concerning for both public health and the sustainability of healthcare systems [18]. In our study, 7.9% of HCWs missed the opportunity to complete the primary vaccination schedule, and 73.7% missed the vaccination opportunity available for the respective period. Additionally, nearly half of the vaccinated HCWs were not within the protective window at the time of infection. These findings highlight the critical importance of improving vaccination uptake among HCWs to reduce individual health risks and break transmission chains. Factors contributing to vaccine hesitancy among HCWs, such as concerns about vaccine efficacy, safety, side effects, misinformation, and lack of knowledge, have been documented in the literature [18,19]. Addressing these barriers through educational programs is essential for protecting HCWs and reinforcing their role as models for public health.

Workforce loss due to COVID-19 has significantly impacted healthcare systems. The results of our study revealed that, due to COVID-19, our hospitals experienced a workforce loss ranging from 7 to 90 days per HCW, amounting to a total of 23,454 days. This duration exceeded the period anticipated by our institution and national health authorities by an average of 1.3 days per HCW and a total of 2,891 days. Maltezou et al. [20] reported that HCWs’ absence durations ranged from 5.82 to 33 days, depending on symptom severity, healthcare system capacity, and pandemic management protocols. Similarly, cumulative workforce loss was reported to increase during periods dominated by more transmissible variants [21]. Our findings align with this, showing a notable increase in workforce loss during the Omicron variant period. Work absences among HCWs due to SARS-CoV-2 infections may be prolonged by psychological factors such as burnout and stress in addition to physical recovery needs [22]. Considering these findings, strategies to enhance the resilience of healthcare systems during future pandemics should focus on effective workload management and support mechanisms.

## 5. Conclusion

HCWs have been disproportionately affected during the COVID-19 pandemic, emphasizing the critical importance of infection control measures. Our study highlights the complexity of infection sources, the need to improve vaccination rates, and the significant impact of workforce loss on healthcare systems. For future pandemics, promoting infection control culture across all healthcare units, addressing vaccine hesitancy, and optimizing shared spaces are essential. These findings would provide valuable guidance for protecting HCWs and ensuring the sustainability of healthcare systems.

**Supplementary material 1:** SARS-CoV-2 Infected Healthcare Worker Follow-up Form

Name-surname	Occupation/Years in occupation
**ID number**	**Unit of Employment at the Time of SARS-CoV-2 Infection**
**Age**	**Time of SARS-CoV-2 Infection (Month/Day/Year)**
**Gender**	**Telephone number**
**COVID-19 vaccination status**	
**Days since the last dose of COVID-19 vaccine**

1. **Questions regarding the source of SARS-CoV-2 infection**
  - Infected while working in the COVID-19 units?
    YES
    NO
  - Was there compliance with the use of personal protective equipment (PPE) if infected while working in the COVID-19 units?
    YES
    NO
  - If not infected while working in the COVID-19 units, was a COVID-19 patient under observation in the unit at the time?
    - YES
    - NO
  - If the answer is ‘YES’ to the previous question, was PPE compliance maintained during contact with the patient?
    YES
    NO
  - Were there other co-workers infected at the same time/within one week?
    YES
    NO
  - If the answer is ‘YES’ to the previous question, was there close contact without surgical masks or social distancing for over 15 minutes?
    YES
    NO
  - Was there a COVID-19 case in the close community (household, etc.) a week before infection?
    YES
    NO
  - If the answer is ‘YES’ to the previous question, was there close contact without surgical masks or social distancing for over 15 minutes?
    YES
    NO
  - Infected while actively working in the hospital? (For those infected during a period of absence due to annual leave, administrative leave, medical report, etc., the NO option should be selected)
    YES
    NO
2. **Questions Regarding Contact Tracing**
  - Within 48 hours prior to infection, how many co-workers in the hospital were in contact for over 15 minutes without surgical mask and social distancing?
  - Risk category of each in-hospital contacts
  - Actions taken for hospital contacts:
  - How many hospital contacts were infected due to this contact?
3. **Questions regarding prognosis and sickness absenteeism**
  1. Was hospitalization required? ............ If yes, number of hospitalization days: ........................
  2. Was ICU admission required? ............. If yes, number of ICU days: ........................
  3. Days between infection detection and return to work: ........................

**Supplementary material 2:** Monthly distribution of the number of SARS-CoV-2 infected healthcare workers

	2020	2021	2022	2023
January	-	40	435	11
February	-	12	362	1
March	-	11	137	-
April	8	45	26	-
May	6	13	31	-
June	31	11	13	-
July	19	8	124	-
August	55	18	76	-
September	111	24	14	-
October	93	28	17	-
November	204	12	53	-
December	54	15	35	-

## Figures and Tables

**Table 1:** Risk categories of HCWs exposed to SARS-CoV-2

	Healthcare worker’s PPE usage status	ContactrRisk
Close contact with a COVID-19 patient/colleague wearing a surgical mask	Did not use a surgical mask or N95 mask, or used a surgical mask in a situation requiring N95 mask	Moderate
	Did not use eye protection	Low
	Did not use gloves and gown	Low
	Used all PPE properly	Risk cannot be assessed
Close contact with a COVID-19 patient/colleague not wearing a surgical mask	Did not use a surgical mask or N95 mask	High
	Used a surgical mask in a situation requiring N95	Moderate
	Did not use eye protection	Moderate
	Did not use gloves and gown	Low
	Used all PPE properly	Risk cannot be assessed

PPE: Personal protective equipments

**Table 2:** Determination of infection sources among SARS-CoV-2-infected HCWs.

	Inappropriate use of PPE during contact with a COVID-19 patient	Contact with a colleague whose SARS-CoV-2 infection is not yet known	Contact with an individual infected with SARS-CoV-2 in the close community outside the hospital
Unidentified source	No	No	No
Community-acquired	No	No	Yes
Hospital-acquired, exposure from patients	Yes	No	No
Hospital-acquired, exposure from colleagues	No	Yes	No

PPE: Personal protective equipments

**Table 3:** Demographic and occupational characteristics of SARS-CoV-2 infected health care workers (HCWs).

Age	36 (±9.6)

Sex, n (%)	
Female	1474 (68.5)
Male	679 (31.5)

Occupation*, n (%)	
Group I	423 (19.7)
Group II	849 (39.4)
Group III	258 (11.9)
Group IV	170 (7.9)
Group V	90 (4.2)
Group VI	148 (6.9)
Group VII	215 (10.0)

Years of professional experience, median (minimum–maximum)	9 (1–44)

Unit of work**	
COVID-19 Units***	285 (13.2)
Others	1868 (86.8)

* Group I: Medical doctors; Group II: Nurses; Group III: Caregivers; Group IV: Cleaning staff; Group V: Pharmacists, psychologists, biologists, dietitians, physiotherapists, laboratory staff, radiology technicians, anesthesia technicians; Group VI: Other technicians, security personnel, waiters, cafeteria staff, kitchen workers, tailors, drivers; Group VII: Managers, engineers, informational technologies staff, officers, secretaries

** Unit of work: The unit where the HCW was working at the time of SARS-CoV-2 infection

*** COVID-19 Units: COVID-19 outpatient clinics, emergency services, COVID-19 inpatient wards, COVID-19 intensive care units.

**Table 4:** Comparison of the source of infection by the unit of work in HCWs infected with SARS-CoV-2 with identifiable sources.

	HCWs in non-COVID-19 units, n (%)	HCWs in COVID-19 units, n (%)	p value
Community-acquired	722 (57.9)	40 (5.2)	<0.001
Hospital-acquired, exposure from patients	71 (5.7)	85 (54.5)	
Hospital-acquired, exposure from colleagues	454 (36.4)	82 (15.3)	

HCW: Healthcare worker

**Table 5:** Expected, actual, and unforeseen workforce loss among health care workers (HCWs) infected with SARS-CoV-2.

	1st period	2nd period	3rd period	Total

SARS-CoV-2-infected HCWs (n)	762	55	1335	2152

Expected workforce loss (days)	10,668	550	9345	20,563

Actual workforce loss (days)				
Total	12,824	768	9862	23,454
Median (minimum–maximum)	14 (14–90)	14 (10–21)	7 (7–21)	7 (7–90)

Unforeseen workforce loss (days)				
Total	2156	218	517	2891
Per number of infected HCWs	2.8	3.9	0.4	1.3

1st period: From April 2020 to October 2021

2nd period: From October 2021 to January 2022

3rd period: January 2022 and onwards

## Footnotes

1 World Health Organization. WHO COVID-19 dashboard [online]. Website https://data.who.int/dashboards/covid19/cases [accessed 26 January 26 2025].

2 World Health Organization (2020). Personal protective equipment for COVID-19 [online]. Website https://www.who.int/teams/health-product-policy-and-standards/assistive-and-medical-technology/medical-devices/ppe/ppe-covid [accessed 11 March 2025].

3 GISAID (2023). hCoV-19 Variants Dahsboard [online]. Website https://gisaid.org/hcov-19-variants-dashboard/ [accessed 11 March 2025].

4 T.C. Sağlık Bakanlığı (2023). COVID-19 Bilgilendirme Platformu [online]. Website (in Turkish) https://covid19.saglik.gov.tr/TR-66301/covid-19-rehberi.html [accessed 13 February 2025].

5 Rambaut A, Loman N, Pybus O, Barclay W, Barrett J, Carabelli A, et al, on behalf of COVID-19 Genomics Consortium UK (2020). Preliminary genomic characterization of an emergent SARS-CoV-2 lineage in the UK defined by a novel set of spike mutations. Virological [online]. Website https://virological.org/t/preliminary-genomic-characterisation-of-an-emergent-sars-cov-2-lineage-in-the-uk-defined-by-a-novel-set-of-spike-mutations/563 [accessed 13 February 2025].

6 Centers for Disease Control and Prevention (2021). CDC updates and shortens recommended isolation and quarantine period for general population [online]. Website https://archive.cdc.gov/#/details?url=https://www.cdc.gov/media/releases/2021/s1227-isolation-quarantine-guidance.html [accessed 10 March 2025].

7 World Health Organization (2019). Value gender and equity in the global health workforce [online]. Website https://www.who.int/activities/value-gender-and-equity-in-the-global-health-workforce [accessed 1 March 2025].
